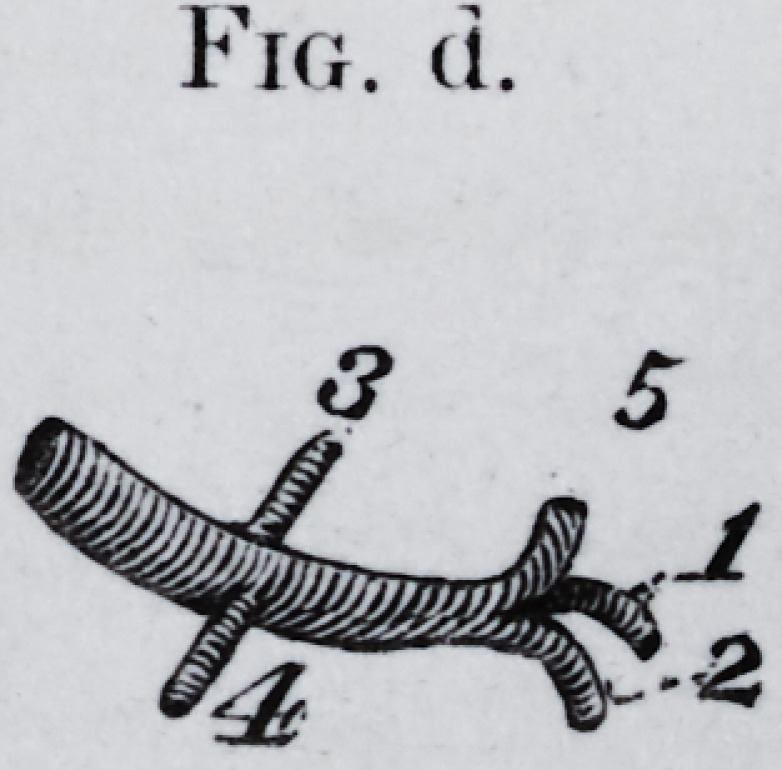# The Salivary Glands

**Published:** 1860-04

**Authors:** Geo. C. De Marini


### ARTICLE VI.

*The Salivary Glands.* By Dr. Geo. C. De Marini.

After mastication, the next chemico-vital process that takes place, is insalivation. This process is performed by means of a series of glands arranged at the sides and underneath the mouth. This series has relations of the greatest importance to the anatomist and dental surgeon. Their function is of particular importance to the animal economy, for, upon their healthy or disordered secretion depends a healthy or diseased state of the mouth. It is to these glands that we purpose to direct our observation in this essay. We will consider them first, in their anatomical relations; secondly, the chemistry of their secretion in a healthy and pathological state, and lastly, the effect of their altered secretions upon the teeth.

The glands which pour their secretions into the mouth are the parotid, submaxillary, sublingual, buccal, labial and palatine. Our description of them will be in the order in which we have mentioned them. The chief glands concerned in insalivation are the three first mentioned. Having corresponding glands on the opposite side of the inferior maxilla they form almost a complete chain around it. They are conglomerate glands with an investing membrane which dips down between their component lobes and lobules. The principal of these is the

### *Parotid Gland.*

This is the largest of the salivary glands, and is larger than all the rest together, and varies in weight from a half to one ounce; it is of a pyramidal form, the apex is inward and the base outward. It seems to be moulded like soft material into the pyramidal space formed by the surrounding parts. It receives its name from its position around the ear. Its coverings are the skin, cellular tissue, superficial cervical fascia, muscular fibres (which are a prolongation of the platysma myoides muscle,) and deep cervical fascia. The deep fascia extends upwards to the zygoma, backwards to the cartilage of the ear, and anteriorly beyond the edge of the masseter muscle. It sends prolongations into the substance of the gland, dividing it into lobes and lobules. The measurements of this gland are from the zygoma to the angle of the jaw from one and a half to two inches, transverse from one and a quarter to one and a half inch, from the integuments to the styloid process, one inch.

On account of the connection of the parotid gland with so many different parts, its relative anatomy is intricate. The anterior surface of the gland extends a little beyond the masseter muscle; this border is grooved to receive the ramus of the jaw; beneath, it extends forwards to the stylo-maxillary ligament which separates it from the submaxillary gland. The posterior surface extends to the cartilaginous portion of the meatus auditorius, and partly curves around it. It also comes in contact with the mastoid process and the posterior portion of the digastric muscle. Superiorly it extends to the zygoma and temporo-maxillary fossa; inferiorly, it fills the space between the angle of the jaw and the border of the sterno-mastoid muscle. The internal surface is very uneven, it fills up the posterior part of the glenoid cavity, surrounds the styloid process, and pterygoid muscles, and comes in contact with the pharynx.

The parotid gland is nourished by the external carotid artery. This artery enters the gland at its lower border, and becoming more superficial as it ascends, it breaks up in the substance of the gland, the final termination of it comes out of the gland near the ear, and ascends, forming the superficial temporal. The external carotid gives off, in the gland the internal maxillary, transverse facial, and posterior auricular.

The veins correspond to the arteries, have similar names and unite to form the external jugular.

The nerves, which are distributed to the parotid gland and integuments, come from the 5th, the portio dura of the 7th pair and cervical plexus: they form constant anastomoses with each other, and subdivide almost ad infinitum. The great auricular divides itself in the substance of the gland, sending off superficial branches and then passes out to the cheek, where it divides into superficial and deep auriculars. The auriculo-temporal arises from the superior maxillary and divides into the superficial temporal and the auricular branch; the latter enters the gland and sends off numerous branches anastomosing with other nerves.

The portio dura of the 7th pair after issuing from the stylo-mastoid foramen, sends off three muscular branches, and then enters the gland, where it divides into the temporo-facial and cervico-facial. These various branches of the portio dura anastomosing with those enumerated, form the plexus called *pes anserinus*. The nerves which form this plexus are the temporo-facial, the auricular branch of the inferior maxillary, and the cervico-facial.

The parotid is the type of the other salivary glands, and the description of the minute anatomy of one will apply with but slight exception to the others. It is a conglomerate gland, of an ash color, which exhibits a granulated appearance upon dissecting off the parotid fascia. It is composed of lobes which again are divided into lobules, and these are connected with each other by areolar tissue, vessels and ducts. The lobes vary in diameter from one-eighth to one-fifth of an inch, and the lobules composing them, measure in diameter from $\frac{1}{60}$ to $\frac{1}{100}$ of an inch. Each lobule consists of numerous closed vesicles, which open into a common duct. This arrangement has been compared to a bunch of grapes upon its stalk. Each grape, with its stem might represent a lobule with its duct, whilst each stem running into a principal stem, and then into the vine, would represent the combination, and course of the ducts.

The wall of each vesicle is formed by delicate basement membrane lined with epithelium and covered with dense capillary net work. Between the lobules we find cellular tissue, in which are found a few lymphatic glands. These lymphatics are sometimes the seat of inflammatory disease and they may become sufficiently enlarged to destroy the gland. The ducts of the separate lobules combining, discharge themselves into a common duct. This arrangement was first described by Needham and afterwards by Stenon, and is now entirely known by the last name. Stenon's duct is then an aggregation of smaller ducts, and leaves the gland at its superior and anterior part, whence it slightly descends and is followed by small filaments of the portio dura nerve. It crosses the masseter muscle, and passing between the fibres of the buccinator enters the mouth at an angle. The mucous membrane of the mouth forms a valve over its orifice, it also lines the duct, and is continued up as far as the terminal vesicle. In mastication, the saliva is forced out partly by the contraction of the fibres of the buccinator and masseter, and partly by the mechanical pressure of the food. The determination of the exact position of Stenon's duct is at times necessary. It may be found by drawing a line from the ala of the nose to the lobe of the ear. To locate the duct, another line must be drawn from the inner canthus of the eye to the anterior and inferior edge of the masseter muscle; the intersection of these two lines is the point at which it perforates the buccinator. The duct of Stenon is composed of mucous membrane and an elastic coat surrounded by areolar tissue. This areolar tissue, as we before said, separates the lobules and contains the ultimate distribution of the blood vessels.

The next salivary gland in its anatomical position and in size, is the

*Submaxillary.*

The description of the minute anatomy of the parotid, will apply to this gland, with the exception that the lobes are somewhat larger than the former, its investing membrane is also less dense. This gland is situated in the supra-hyoid region, in the anterior part of the submaxillary angle of the neck. It is of an irregular form, and weighs about two drachms. Its relations with the surrounding parts vary according to the position of the head and lower jaw. When the lower jaw is depressed it lodges in a space, where it is almost hidden by the inferior maxilla. When the head is thrown back it becomes much more external and seems to belong more to the supra-hyoid than to the submaxillary region. Its coverings are, integument, platysma myoides, deep cervical fascia, and when the chin is depressed it is protected by the lower jaw. It lies upon the mylo-hyoid, hyo-glossus and stylo-glossus muscles, a part of the gland passing beneath the posterior border of the mylo-hyoid. Posteriorly it is separated from the parotid by the submaxillary ligaments and anteriorly it is separated from the sublingual by the mylo-hyoid muscle.

The arteries of this gland come from the facial and lingual. The facial lies in a groove at the posterior and upper border. The nerves are derived from the submaxillary ganglion, from the mylo-hyoid branch of the dental and partly from the sympathetic. The excretory duct of the submaxillary gland is called the duct of Wharton.

Like Stenon's duct it is formed by a union of the small ducts of the lobules. The duct leaves the gland at its anterior extremity and is directed obliquely upward and inward, parallel with the hypoglossal and lingual nerves. It first passes between the mylo-hyoid and hyo-glossus muscles, and then between the genio-hyo-glossus and sublingual gland; at this point it then passes between it and the mucous membrane of the mouth, it perforates the mucous membrane and opens by a narrow orifice upon a papilla, behind the incisor teeth. The duct is almost two inches long, is much thinner than Stenon's, and yet much larger in proportion to the size of the gland.*

### *The Sublingual Gland*

Is the third and smallest of the salivary glands. Its general anatomical characteristics are similar to the others. Its weight about one drachm. It is situated directly beneath the mucous membrane of the mouth on each side of the frœnum linguæ, it is here also in contact with the inner surface of the jaw near the symphysis. Interiorly it is in relation with the genio-hyo-glossus muscle from which it is separated by the lingual nerve, and also with Wharton's duct. Its anterior extremity is in contact with its fellow of the opposite side. Behind it touches the deep part of the submaxillary gland. Its excretory ducts, sometimes called the ducts of Rivinus, open into the mucous membrane of the mouth along the sublingual crest. Others again open directly into Wharton's duct. The arteries of this gland come from the submental and sublingual. The veins have the same name. Its nerves are numerous branches of the gustatory.

### *The Labial, Buccal and Palatine.*

The next glandular series connected with insalivation, are found under the mucous membrane of the lips, cheeks and palate. These minute glands form a layer between the mucous and muscular layer of the structure forming the oral cavity. They are different from the muciparous follicles and are true salivary glands. They are distinct, spheroidal glands, and have secretory ducts opening upon the mucous membrane of the mouth ; the orifices of their ducts produce a perceptible elevation of the mucous membrane. These congregations of salivary glands are called

* For some further particulars relative to the anatomy of this gland, see the "Description of Plates."

as above. The nature of their secretions will be considered under the head of saliva.

The great function of all these glands is the secretion of saliva.

Our attention will now be directed to this secretion, and will embrace its chemical and physiological action; we will also notice some of the pathological changes it undergoes, and the effect of those changes. The saliva differs in density and manner of discharge in the various glands.

The secretion of the parotid gland discharges chiefly during mastication or irritation of the mucous membrane of the mouth, while the submaxillary and sublingual are continually pouring out. The saliva of the parotid then has been named the "saliva of mastication."

The secretion of the other glands is much more tenacious and seemed to serve the purpose only of lubricating the food and facilitating deglutition. It has been called the "saliva of deglutition." The differences mentioned concerning these secretions, was discovered by alternately tying the excretory ducts of the salivary glands of animals. That the functions of the two varieties of saliva are as stated, is confirmed by comparative anatomy. Animals which masticate their food have large parotids, and those that merely lubricate their food before swallowing it, have large submaxillary and sublingual glands.

### *Chemistry of the Saliva.*

It is difficult to obtain a sufficient quantity for analysis, and to get it pure and free from epithelial scales. It should be obtained from fistulas. Saliva is a clear, colorless and viscid fluid. Upon standing, it separates into an upper stratum of clear fluid, and at the bottom another stratum of denser fluid, and of course not so transparent. The lower one contains epithelial scales. The specific gravity varies from 1004 to 1009. The reaction of saliva is usually alkaline, but this is subject to variation. Analyses of saliva have been made by most of physiologists.

We give two of the most reliable:

Dr. Wright, *of London.*

| | |
|---|---|
| Water, - - - - | 988.1 |
| Ptyaline, - - - | 1.8 |
| Fatty acid, - - - - | .5 |
| Chlorides of sodium and potassium, | 1.4 |
| Albumen with soda, - - | .9 |
| Phosphate of lime, - - | .6 |
| Albuminate of soda, - - | .8 |
| Lactates of potash and soda, - | .7 |
| Sulphocyanide of potassium, - | .9 |
| Soda - - - - - | .5 |
| Mucus with some ptyaline - | 2.6 |
| | 998.8 |

Bidder and Schmidt.

| | |
|---|---|
| Water, - - - - | 955.16 |
| Organic matter, - - | 1.34 |
| Sulphocyanide of potassium, - | 0.06 |
| Phosphates of soda, lime and magnesia, | .98 |
| Chlorides of sodium and potassium, | .84 |
| Mixture of epithelium, - | 1.62 |
| | 1000.00 |

The variations in the results of analyses of saliva may be on account of changes in the secretion, for it is certain that the secretions vary according to the state of general health, the condition of the brain and nervous system, and perhaps the character of the food.

The presence of the sulphocyanide of potassium in the saliva is somewhat remarkable, as it exists in none of the other secretions. It has been since its first discovery, a question whether it is a normal or pathological element of the saliva. Mr. Longet in the "Comptes Rendus" in

1856, gives very conclusive arguments in favor of its normal existence. His chief arguments are that it is found in the secretions of all of the large salivary glands. That it is found universally when the proper tests are applied. That, where it has not been discovered, a proper degree of concentration of the saliva was not obtained, and that it exists without regard of changes in the teeth. Its effect upon the animal economy and the reason for its presence only in this secretion have not been ascertained.

It has been asserted by some physiologists, and among them by Wagner, that the saliva has no important digestive function. His conclusion seems to be mainly drawn from the fact that food placed directly into the stomach was as readily digested as if it had been mixed with saliva. Experiments upon it, isolated from other fluids of the body lead to a different conclusion. Although it must be said that it has not been positively proved to be absolutely necessary to digestion.

The results of experiments made upon saliva out of the body, are briefly these. It was found to convert starch into sugar; the transformation being so complete that iodine would not detect the least trace of starch in the mixture. These effects required the combined fluids of the mouth and did not produce the same effects when the saliva of the larger salivary glands alone was taken.

The test for detecting starch is to add iodide of potassium, and afterward nitric acid. Iodine will always show a blue color when acting upon starch. In this case the nitric acid unites with the soda, setting the iodine free.

To find whether sugar exists in a mixture, Trommar's test is the most delicate. It is composed of sulphate of copper, tartrate of potassa and potassa fusa; these are dissolved in water. When added to the liquid to be tested, they impart a blue color to it which upon the mixture being boiled, turns to yellow if sugar be present, this substance reducing the oxyd of copper to the red suboxyd.

Dr. Wright, of London, found that this property of converting starch into sugar was increased by impregnating it with oxygen. Saliva seems also to have a fermentative property. When mixed with meat and bread, and allowed to stand two days, it produced vinous fermentation. The same material when mixed with water and kept at the same temperature were found to decompose more readily.

These are the only positive chemical properties that saliva seems to have, and it may be doubted, until more light is thrown on the subject, whether, with this exception, saliva does more in the animal economy than to lubricate and soften the food, except that we admit that its antiseptic property is of importance. Experiments made upon animals with the salivary ducts tied, showed that the food becomes sour after being retained in the stomach a certain length of time. This, of course, would retard digestion and ultimately seriously affect the general health.

The characteristic ingredient of saliva is ptyaline; its presence is invariable and gives to saliva its peculiar smell. Ptyaline may be obtained as follows: Filter the saliva and treat the residuum with sulphuric ether, this will then contain the fat and ptyaline, evaporate and the dissolved fat is volatilized with the ether, leaving ptyaline. When obtained it is a solid, adhesive and yellowish substance, and has the characteristic odor of saliva. It may be preserved for some time without decomposition.

We will now leave the chemistry of healthy saliva and consider the morbid changes this fluid undergoes, only observing those changes which affect the teeth. Saliva may contain, as an abnormal constituent, any of the secretions of the body, and also any of its chemical constituents. This fact would vary the reaction of saliva, and thus we find it at times quite acid and at others unusually alkaline. It is evident that these two changes would produce an action upon the teeth, and we leave other morbid conditions and turn our attention only to these.

*Acid Saliva.*—The acids with which saliva has been found impregnated, are acetic, lactic, hydrochloric, oxalic and uric. The presence of these acids is invariably due to an altered state of the general system. Among the causes are fevers, especially eruptive fevers, rheumatism, gout, diabetes, and a disordered state of the digestive organs; the latter is no doubt the most frequent cause. The use of certain food and drinks will also produce acidity of this secretion.

The effect of acid saliva upon the teeth is active corrosion. The destructive power of acids upon the teeth is too well known to require repetition; the acid combines with the alkaline substance of the teeth and destruction of the enamel and bone takes place with fearful rapidity. It is a well known fact that if a tooth is placed in dilute acetic acid, it will in a short time entirely disappear. As a cause of caries, the skillful dentist should never overlook the state of the saliva, and a mild medical treatment might be instituted with his operations upon the teeth.

*Alkaline Saliva.*—This condition depends usually upon excess of soda; there is also an excess of albumen and the usual properties of the secretion are diminished. The causes of alkaline saliva are frequently depending upon disturbances of the nervous system; neuralgia, toothache, mental emotions, mania and epilepsy, are some of the causes of it. Ammonia is sometimes also a cause of alkaline saliva; but this is unusual, and when it exists the saliva has a most unpleasant taste and smell, it is diminished in quantity and becomes very viscid.

To the dentist an important variety of alkaline saliva is the presence of calcareous matter. This, of course, is one of the causes of alkaline saliva, and is the formative principle or element of salivary concretions.

These concretions are found in the tartar of the teeth and in salivary calculi. The chemical ingredient which is found to excess in these deposits, and in the saliva that causes them, is the phosphate of lime. Its normal proportion is 6 parts in a 1000, but when an excess over this takes place, accumulation of calcareous matter is the consequence.

Where there is an excess of this ingredient in saliva, the secretion shows it without the necessity of chemical analysis; it has a whitish appearance, different from the transparent of ordinary normal saliva, and its reaction is very decidedly alkaline.

The effects of calcareous saliva upon the teeth is known to all. It deposits its excess of earthy constituents around the teeth at their junction with the gums and fills any interstices that may exist in them. Any roughness or obstruction becomes a nucleus of deposit.

The teeth may be regarded as separate nuclei of deposit, but a collection in the salivary ducts is less easy of explanation. Fortunately they are rare, and when found, may be traced to some obstructing cause. Dr. Piggot gives an analysis of a calculus found in the duct of Stenon of a horse, which may be regarded as a chemical type of this class of calculi as found in the herbivora. The nucleus in this case was found to be a splinter of pine wood.

The result of analysis was

| | |
|---|---|
| Water, | 1.260 |
| Organic matter, | 8.350 |
| Lime, | 49.572 |
| Magnesia, | 2.820 |
| Alkalies, | .816 |
| Carbonic acid, | 31.702 |
| Phosphoric acid, | 2.320 |
| Silica, | .330 |
| Chlorine, | .747 |
| Sulphuric acid, | 2.019 |
| | 99.938 |

The excess of lime in this analysis is rather greater than is found in the analysis of salivary concretions in the human body, but may be taken as a standard on account of its completeness, by which we can more easily estimate the relative proportions of salivary calculi in other animals. The tartar of the teeth is the commonest form of salivary concretion. We give the analysis of this substance, made by Pepys and Dr. Dwinelle, of Cazenovia, New York.

| | Pepys. | Dwinelle. |
|---|---|---|
| Phosphate of lime, . . | 35 | 60 |
| Carbonate of lime, . . . | | 14 |
| Fibrin, or cartilage, (?) . | 9 | |
| Animal matter and mucus, | | 16 |
| Animal fat, or oil, . . | 3 | |
| Water and loss, . . | 3 | 10 |
| | 50 | 100 |

Ptyalin has also been found as an element in salivary calculi. Its presence is not invariable.

Having concluded the anatomy of the salivary glands, the physiology and chemistry of the saliva, we will make a brief notice of the diseases that the glands are liable to. For this purpose we will again take the parotid as a pathological type. The lymphatic glands may become diseased and enlargement to an extraordinary degree may be the result. They have become sufficiently enlarged to cause absorption of the gland itself.

*Parotitis* is the most ordinary form of disease of this gland. A similar inflammation sometimes affects the other glands.

*Induration* may take place as the result of inflammation and depends upon an interstitial deposit of lymph.

*Abscesses* are of frequent occurrence and usually form under the internal fascia, and are the cause of intense pain.

*Scirrhus* is the gravest disease which the salivary glands are liable to. Its existence is indicated by an irreg-

Plate 2.

PLATE 1.

PLATE 3.—FIG. a.

FIG. b.

FIG. c.

FIG. d.

ular and intensely hard swelling with clefts and globular projections on its surface, accompanied by lancinating pains, and the ducts have a firm, round feeling under the finger.

*Sarcoma.* In this form of disease the gland is also enlarged, but the growth is more rapid than the last, and it is less lobulated and has not that firm feeling of the former.

Beside these diseases we may find degeneration of tissues, as fatty and encephaloid degenerations, tumors in the substance of the glands, fungous growths, &c., &c.

With these very imperfect and hastily gathered observations I leave the subject. Not that I have by any means completed it, but collecting notes and verifying anatomical plates by dissections, consumed the time that might have been devoted to a better arrangement of my materials and a clearer statement of facts.

### *Description of Plates.*

### PLATE I.

Is a copy of the illustration of the glands from Gray's Anatomy. As his plate has in some respects a distorted appearance and does not represent the natural proportion of the glands, I made a dissection, which Plate II represents.

### PLATE III.

*Fig. a*, represents the right submaxillary gland, as it first appears on dissecting, of natural size and invested by its membrane.

*Fig. b.* The same taken out, divested of its membrane and seen from above. I find it is composed of six distinct lobes.

*Fig. c.* The same with the lobes stretched apart to show their relative size and form. This figure also shows the principal ducts, arteries and veins. No. 6 is the part which passes beneath the posterior border of the mylohyoid. The lobes numbered 1, 2, 3, 4 and 5, are nourished by a branch of the facial artery, whilst lobe 6 receives a branch from the lingual.

*Fig. d.* The facial artery of natural size as it appears injected. The branches go to the lobes with corresponding numbers.

## Figures and Tables

**Figure f1:**
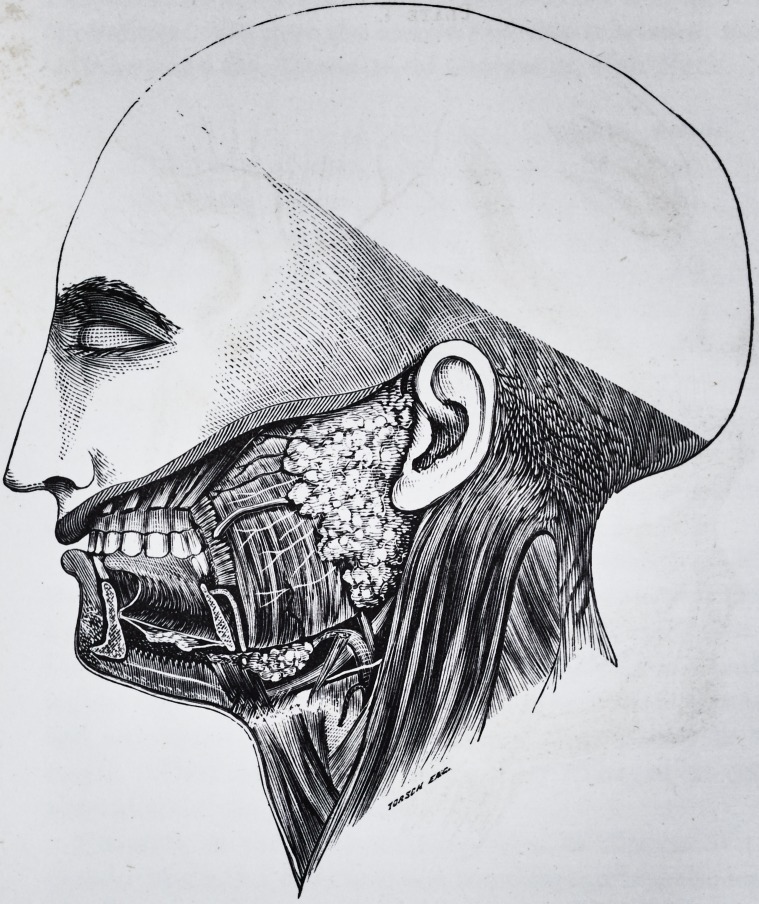


**Figure f2:**
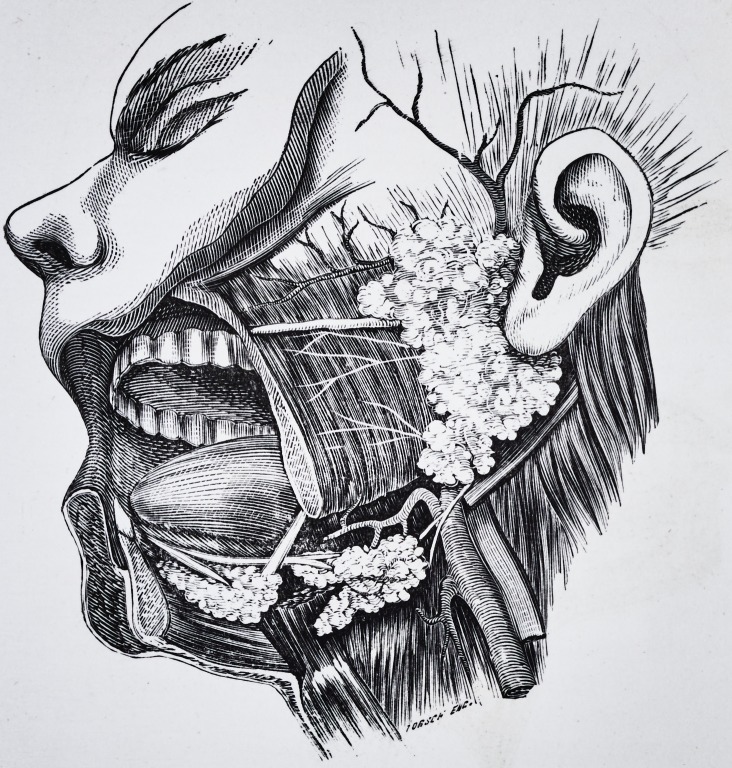


**Plate 3.—Fig. a. f3:**
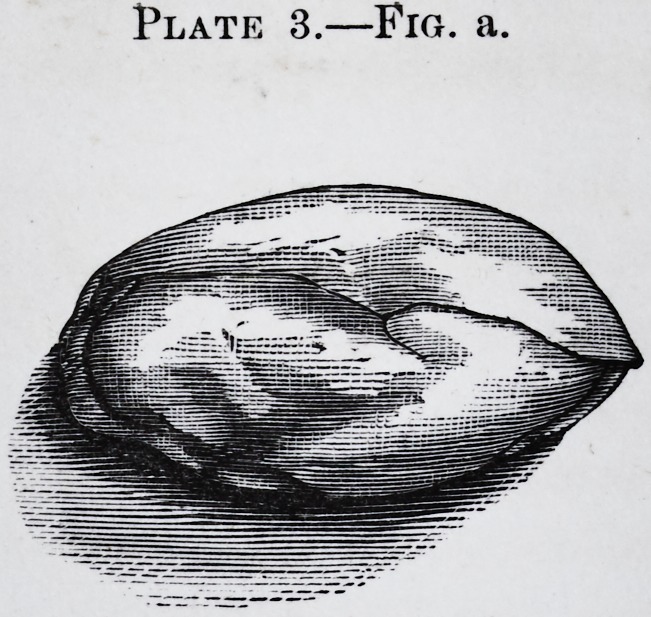


**Fig. b. f4:**
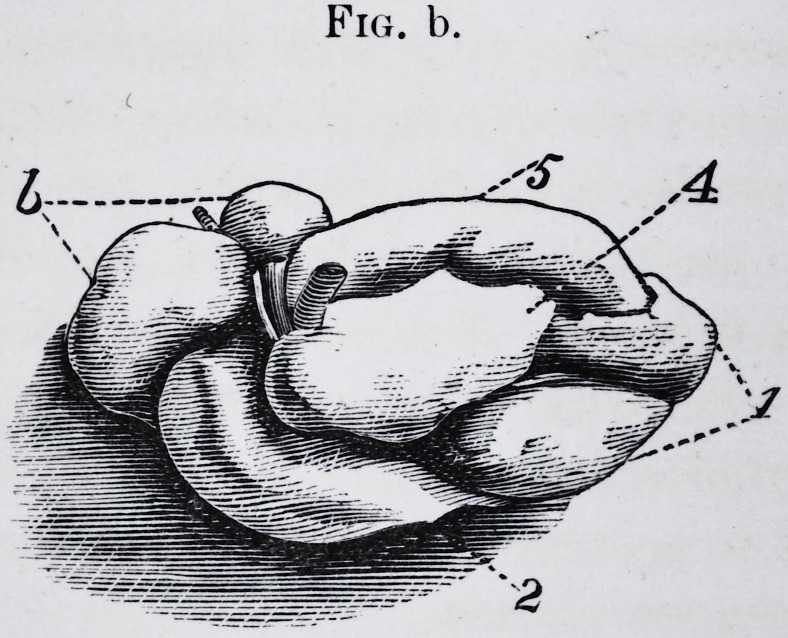


**Fig. c. f5:**
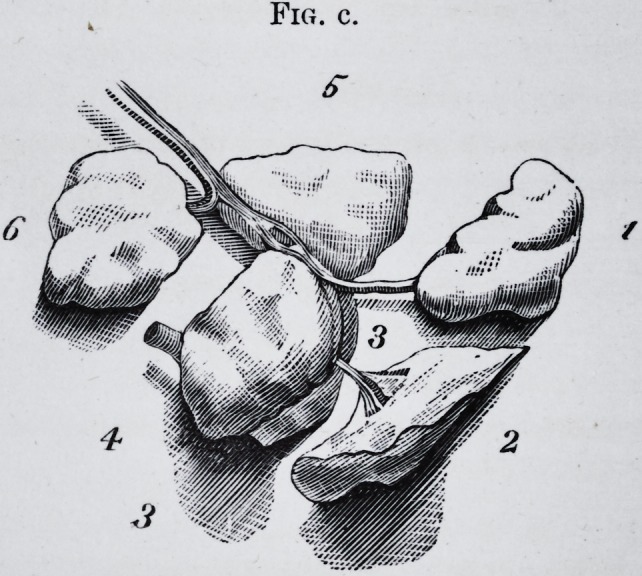


**Fig. d. f6:**